# Biological Psychiatry, Research And Industry

**DOI:** 10.4103/0973-1229.32155

**Published:** 2007

In this section, we look at how the biological paradigm shift in psychiatry has been aided and abetted by industry for serving its own needs; which stymies other promising approaches; but which, nonetheless, can serve to advance biomedicine if checks and balances are in place.

## Industry, Biological Psychiatry And Non-pharmacological Advance

The larger issue of benefit to society also concerns us when we realize that industry sponsorship is mainly for potential medications, not for trying to determine whether there may be non-pharmacological interventions that may be equally good, if not better.

…a lack of balance in research activities, with a focus mainly on potential medications, is likely to divert talented researchers from the pursuit of profound scientific questions or divert them from the pursuit of questions without market relevance but with an aspect of public good. A company has little incentive to support trials evaluating whether inexpensive, off-patent drugs or whether non-pharmaceutical interventions, could replace their profitable patented drug (Baird, 2003)

This is the reason why methods like yoga, psychotherapy, meditation, non-medicated non-mechanised relaxation will not find industry sponsors readily and may never be proved useful apart from anecdotal reporting.

In which case to expect industry sponsorship to develop a larger therapeutic armamentarium, especially non-drug based, is wishful thinking. Moreover, non-pharmacological treatment procedures may not get desirable funding. This may not be as much of a problem in other branches of medicine as in psychiatry, wherein non-pharmacological interventions like psychotherapy still hold promise of therapeutic relief.

If we do not see rigorous experimental research in psychotherapy or other non-drug modalities to the extent that we should, let us be careful before blaming the researchers for it. Where are the funds? Also, let us note that behind the great thrust towards Biological Psychiatry, neuro-physiology and neuro-pathology, is not just the commitment of a dedicated band of researchers out to reach the truth of psychiatric diseases. That is there, of course, and is laudable. Equally important is the fact that Biological approaches get industry sponsorship and furthering it legitimizes industry's role and serves its interests. Biological approaches find neuro-physiological and biochemical correlates of behaviour and abnormalities and strengthen the case for psychopharmacological approaches as the mainstay of treatment.

This is not to deride the legitimate growth of Biological approaches, it is only to understand why it is preferred over other approaches for funding and has occupied center-stage in research today. And this state of affairs obtains in other branches of medicine too. If the psychotherapeutic approach is neglected because there is no possibility of benefit to industry, the psychosomatic approach to medicine is neglected because it is equally of little benefit to industry growth. So findings, for example, of heightened stress and hostility as aetiologic in coronary heart disease get token mention, whilst sophisticated investigative and therapeutic procedures and related medications get highlighted. What makes for sound ethical practice hardly makes for sound business sense for both the researcher and industry.

## The Clear Picture

So, the picture is very clear. Industry is not in medicine or psychiatry, for comprehensive patient welfare. It will further only those researches/researchers that earn it profits. Hence biological research flourishes. Psychological and sociological researches have to fend for themselves, which is not at all easy. So they languish and the smart guys who want to rise realize it quick and devote their attention to biological approaches. The lesser minds or the very devoted, do to the rest, but they are working against the prevailing current and must persevere against many odds. Most are pragmatic enough to read the writing on the wall and fall in line. Psychological research reduces or is done by the less bright minds and this is cited as evidence that psychological approaches do not have as good empirical proof of utility as the biological ones have. Psychiatric practitioners start priding themselves in learning the most recent advances in biology and pay lip service, if at all, to the psychological ones. Even textbooks may mention psychological approaches, but it is more out of trying to give a complete picture rather than doing justice to them. Neither do the people doing research there do justice to the approach, for they are not backed with adequate funds or with great following or even with very bright minds.

## Paradigm Shift Towards Biological Psychiatry

The pervasive influence of industry on psychiatric practice, training and research has come in for some careful scrutiny. Healy and Thase talk of how academic psychiatry is for sale because of pharma influence (Healy and Thase, 2003). Perlis *et al.* (2005) have studied the extent and implications of industry sponsorship and financial conflict of interest in psychiatric clinical trials. They report that author conflict of interest appears to be prevalent among psychiatric clinical trials and is associated with a greater likelihood of reporting a drug to be superior to placebo. This is their finding:

Among 397 clinical trials identified, 239 (60%) reported receiving funding from a pharmaceutical company or other interested party and 187 studies (47%) included at least one author with a reported financial conflict of interest. Among the 162 randomized, double-blind, placebo-controlled studies examined, those that reported conflict of interest were 4.9 times more likely to report positive results; this association was significant only among the subset of pharmaceutical industry-funded studies (Perlis *et al.*, 2005).

Shooter (2005) tries to strike a balance, even when asking a provocative question about academia-industry relationship: if it amounts to dancing with the devil. The two differing views of Brodkey and Mohl on industry influence of psychiatry resident training are interesting. Brodkey (2005), while describing and examining the role of the pharmaceutical industry in the teaching of psychopharmacology to residents and medical students so as to make recommendations for changes in curriculum and policy based on these findings, is firmly of the view that the medical profession must reassert control of medical education and draw a firm barrier between commercial and professional pursuits. Mohl (2005) is more accommodative when he accepts that:

…the high visibility of the pharmaceutical industry appears to be a permanent fixture of the world our residents will enter upon graduation… Our trainees must be prepared to cope with its (free market economy and its ethos) opportunities, enticements and seductions… Total prohibition infantilizes our trainees. With careful understanding of the complex nature of the physician/pharmaceutical industry relationship and detailed case-by-case analysis of the dimensions of agenda and control, one can-indeed, one must seek drug company resources to maximize training opportunities. But we should have no illusions: psychiatric educators must be constantly watchful and mindful of the pitfalls along this road. Neither obliviousness to the dangers nor total avoidance serves our trainees′ (Mohl, 2005).

Goel (2004), in a keynote address, notes how the unholy pharma-psychiatry nexus manifests in myriad ways. According to him, its effect can be blunted by three means: first, drugs prescribed only by their generic/pharmacological names; second, pharmaceutical industry-supported research funded in a transparent manner - funds routed through the institution where such research is conducted and the relevant information available in the public domain; and third, Conferences and other academic events also funded in a transparent manner, the advertisements restricted to a dedicated brochure, wherein full fiscal details are disclosed (ibid).

Bola (2003) pinpoints that as the field moves more towards implementation of evidence-based practice guidelines, the importance of removing bias remains central to providing optimal clinical care. And Tyrer (2003), firmly tongue-in cheek, on assuming editorship of the *British Journal of Psychiatry*, labels academic psychiatry and drug-industry relationship reflections as a ding-dong debate.

### Industry And Paradigm Shift

All these issues are important and need detailed discussion in their own right. But what we want to highlight here is the fact that in the paradigm shift towards biological psychiatry, the role of industry sponsorship is not overt but probably more pervasive than many have realized or the right thinking may consider good for the health of the branch in the long run.

Ask yourself a simple question: Why should industry sponsor psychological research? Why? And why should industry *not* sponsor biological research? Why not? Which of the two will give rise to drugs? Marketable products? How will the profits pour in? The answer is simple enough.

## Will This Situation Change?

This situation is hardly likely to change unless major catastrophes occur due to the biological approach or a great contrary force due to a modern Freud or Adler makes its mark. This appears a tall order at present, since the biological approach is not without merit and is based on sound empirical grounds. On the other hand, the psychological does get quite some lip sympathy from the right thinking, but not the type of committed research that could resurrect it into a force to reckon with. But we can never say what the future holds, for trends can change dramatically with one great leader. Or one major catastrophe. And trends can start changing with the realization that this thought can bring into people like you and us.

All this, provided we have the long-term benefit of our discipline at heart. Well, if it is swimming with the tide or dancing with the porcupine and enjoying the fruits of such labours that we seek, then what situation obtains at present is but appropriate.

Are you ready to swim against the current?
